# Crystal growth of La_2/3-*x*_Li_3*x*_TiO_3_ by the TSFZ method

**DOI:** 10.1098/rsos.181445

**Published:** 2018-12-05

**Authors:** Yuki Maruyama, Shiho Minamimure, Chinatsu Kobayashi, Masanori Nagao, Satoshi Watauchi, Isao Tanaka

**Affiliations:** Center for Crystal Science and Technology, University of Yamanashi, 7-32 Miyamae, Kofu, Yamanashi 400-8511, Japan

**Keywords:** crystal growth, travelling solvent floating zone method, anisotropic ionic conductivity, Li ion battery

## Abstract

Double-perovskite-type La_2/3-*x*_Li_3*x*_TiO_3_ (LLT) crystals were grown by the travelling solvent floating zone (TSFZ) method. When the floating zone (FZ) crystal growth method was applied, the La_2_Ti_2_O_7_ phase was deposited as an inclusion in the initial growth region. Using the TSFZ crystal growth method, however, inclusion-free LLT crystals were obtained for a 10 mol% La_2_Ti_2_O_7_-poor composition solvent relative to the stoichiometric LLT crystals. The molten zone was initially unstable as a result of habit plane formation during the crystal growth; however, the molten zone was stably maintained for a long period of time by decreasing the feed rate compared with the growth rate. Hence, LLT crystals of approximately 5 mm*φ* and 37 mm in length were obtained. The anisotropic ionic conductivity of the crystals annealed in air was *σ*[110]/*σ*[001] ≈ 3, with *σ*[110] = 1.64 × 10^−3^ S cm^−1^ and *σ*[001] = 5.26 × 10^−4^ S cm^−1^. LLT single crystals are candidates for high-performance solid-state electrolytes in all-solid-state Li ion batteries.

## 1. Introduction

Conventional Li ion batteries incorporating liquid electrolytes are widely used in electric and portable devices; however, these batteries are associated with various potential problems such as electrolyte leakage and fire. By contrast, all-solid-state Li ion batteries with a solid electrolyte are highly safe, being non-flammable and having zero leakage. Moreover, all-solid-state Li ion batteries offer significant advantages over conventional batteries, such as thermal stability and large potential windows allowing the use of high-voltage cathode materials and/or metallic Li anodes [1–3]. Thus, all-solid-state Li ion batteries are promising as next-generation storage batteries.

Inorganic solid electrolytes comprise sulfide and oxide solid electrolytes. Sulfide solid electrolytes are reported to possess high ionic conductivities (*σ*) of 10^−2^ S cm^−1^ comparable to those of liquid electrolytes [4]. However, sulfide solid electrolytes are reactive to moisture in the atmosphere. Oxide solid electrolytes such as perovskite structures, e.g. La_2/3-*x*_Li_3*x*_TiO_3_ (LLT) [5–8]; Na super ionic conductor (NASICON) structures, e.g. Li_1+*x*_Al*_x_*Ti_2-*x*_(PO_4_)_3_ [9,10]; and garnet electrolytes, e.g. Li_7_La_3_Zr_2_O_12_ [11,12], have been extensively studied because of their high chemical stability and high *σ*. It has been also reported that the *σ* of perovskite-type ABO_3_ is improved by the substitution of other elements on the A- or B-sites, such as Ln_1/2_Li_1/2_TiO_3_ (Ln = La, Pr, Nd, Sm) [13], R_1/4_Li_1/4_TaO_3_ (R = La, Nd, Sm, Y) [14,15] and LiSr_1.65_M_1.3_M’_1.7_O_9_ (M = Ti Zr, M’ = Nb, Ta) [16–18]. Among them, LLT is a promising electrolyte because of its high *σ* [5].

It is known that the LLT crystal structure depends on the Li concentration [7,19]. LLT (*x* = 0.167) is a cubic perovskite structure (space group: *Pm3m*), whereas LLT (0.03≦x≦0.167) is a tetragonal double-perovskite structure (space group: *P4/mmm*). Double-perovskite-type LLT is composed of alternately stacked La-rich and -poor layers along the *c*-axis [20]. The *σ* found in LLT (*x* = 0.117), i.e. approximately 10^−3^ S cm^−1^ at room temperature, is the highest yet reported for Li concentrations [6,7]. Thus, double-perovskite-type LLT has been identified as having the most promising structure for use in an Li ion conductor.

To date, a sintered body of the ionic conductor has been used for oxide solid electrolytes. However, the *σ* of a solid electrolyte obtained using powder decreases with the presence of grain boundary resistance [21,22]. By contrast, a solid electrolyte based on a single crystal has no grain boundary and high *σ* is expected. To advance this field, LLT single crystals are required for experiment to clarify their basic physical properties (e.g. their anisotropic ionic conductivity) and to apply a substrate for application in high-performance all-solid-state batteries. Inaguma *et al*. reported the growth of LLT single crystals with dimensions of 3 × 2 × 1 mm^3^ using the floating zone (FZ) method, and discussed their *σ* values [23]. Moreover, Varez *et al*. have reported the growth of LLT fibre single crystals using the laser FZ method [24]. However, large single crystals have not yet been obtained. The end La_2_Ti_3_O_9_, i.e. the end member of La_2/3-*x*_Li_3*x*_TiO_3_, melts incongruently to La_2_Ti_2_O_7_ and a liquid at 1660°C, according to the phase diagram of the La_2_O_3_-TiO_2_ system [25]. It is likely that LLT is also an incongruent melting compound. The travelling solvent floating zone (TSFZ) method, which involves FZ growth using a solvent, is a powerful crystal growth technique for an incongruent-melting compound and solid solutions such as Y_3_Fe_5_O_12_ and La_2-x_Sr_x_CuO_4_ superconductors [26–28].

In the present study, the incongruent-melting behaviour of LLT is confirmed using the FZ method, and larger LLT single crystals are grown by the TSFZ method. The solvent composition for TSFZ growth is determined by clarifying the inclusion phases in crystals grown by the FZ method. The TSFZ growth conditions, such as the solvent composition and feed rate, are then optimized for large LLT single-crystal growth. Finally, the anisotropic ionic conductivity of the LLT grown crystals is clarified.

## 2. Experimental procedure

La_2_O_3_ (Rare Metallic Co., Ltd; 99.99%), Li_2_CO_3_ (Rare Metallic Co., Ltd; 99.99%) and TiO_2_ (Rare Metallic Co., Ltd; 99.99%) were used as starting materials, being weighed at the La_0.55_Li_0.35_TiO_3_ (*x* = 0.117) stoichiometric composition and mixed with ethanol. The mixed powder was calcined at 800°C for 1 h in air. Then, the powder was ground, before being calcined at 1100°C for 12 h in air. The calcined powder was formed into a cylindrical shape by rubber pressing. The resultant rod was then sintered at 1300°C for 12 h in air and used as a feed rod for crystal growth. Solvents with TiO_2_-excess or La_2_Ti_2_O_7_-poor compositions relative to the LLT stoichiometric composition were prepared using the same procedure as feed rod preparation.

For crystal growth, an infrared convergent heating image furnace (Crystal Systems Corporation, FZ-T-4000-H) equipped with four 300 W halogen lamps was used. The crystal growth was conducted under Ar gas flow conditions. The feed and growth rates were 3–5 and 5 mm h^−1^, respectively. The rotation rates of the feed rod and the grown crystal were 8 and 40 r.p.m., respectively. The grown crystals were annealed at 1000°C for 20–30 h under the oxygen gas flow.

The precipitated phases in the grown crystals were observed using an electron probe micro-analyser (EPMA, JEOL JXA-8200). The crystals were cut along the growth direction and the cut surfaces were mirror-polished. Elemental mapping images of La and Ti in the mirror-polished samples were measured using the EPMA. Also, the concentrations of La and Ti in the grown crystals were determined by a quantitative analysis using a La_2_Ti_2_O_7_ single crystal as a standard sample, and Li concentration 3*x* in the composition La_2/3-*x*_Li_3*x*_TiO_3_ was estimated using the atomic ratio La/Ti. The crystallographic axis was identified using the back-reflection Laue X-ray diffraction method and X-ray diffraction (XRD, Bruker Discover D8 with a 2D detector). The Li ion conductivity of the samples was measured by an AC complex impedance method. Au-Pd thin film was sputtered on both sides of the sample surface to maintain the electrode ohmic contact. The complex impedance was measured using an inductance-capacitance-resistance (LCR) meter (Iwatsu Electric Co., Ltd; PSM 1700) in the frequency range of 1 Hz to 10 MHz at room temperature.

## 3. Results and discussion

### 3.1. Crystal growth by the floating zone method

**Figure 1.**
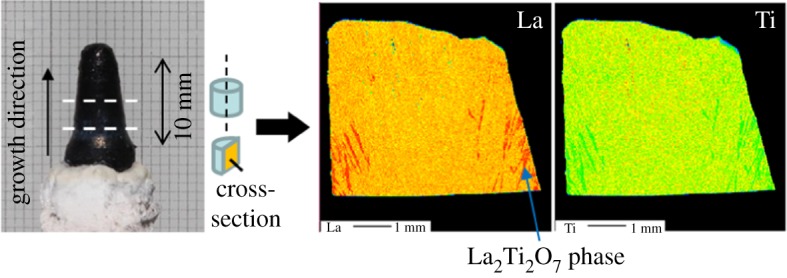
Photograph of crystal grown by the FZ method and mapping images of La and Ti within crystals.

Figure 1 presents a photograph of a crystal grown by the FZ method, along with mapping images of the cross-section of the grown crystal. Crystals of approximately 4 mm*φ* and 11 mm in length were obtained. The crystals were black because of oxygen deficiency due to the growth in Ar gas. During the application of the FZ crystal growth method, it was difficult to maintain a stable molten zone because of inhomogeneous melting of the feed rod. The EPMA analysis indicated that the La_2_Ti_2_O_7_ phase was deposited as an inclusion in the initial growth region. This result indicates that LLT is an incongruently melting compound, because the La_2_Ti_2_O_7_ phase was deposited as a primary phase from the LLT melt. By contrast, the TSFZ method was expected to be effective for LLT crystal growth as it can suppress deposition of La_2_Ti_2_O_7_ inclusions and decrease the growth temperature to reduce Li evaporation from the molten zone.

### 3.2. Solvent composition optimization for travelling solvent floating zone growth

**Figure 2.**
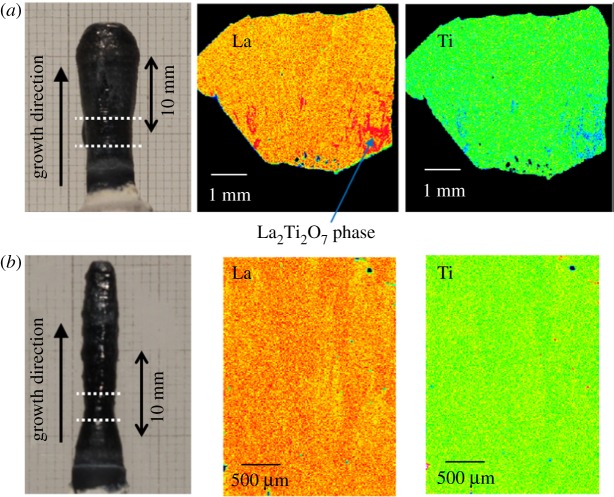
Photographs and La and Ti concentration mapping images for crystals grown by the TSFZ method with (*a*) 5 and (*b*) 10 mol% La_2_Ti_2_O_7_-poor solvent.

During crystal growth by the FZ method, the LLT was found to melt incongruently to the La_2_Ti_2_O_7_ phase and a liquid. In the next stage of this study, we attempted growth of LLT crystals by the TSFZ method, using solvents with TiO_2_-excess or La_2_Ti_2_O_7_-poor composition relative to the LLT stoichiometric composition. From the phase diagram of the TiO_2_–La_2_O_3_ system [25], the composition of the liquid coexisting with the La_2_Ti_3_O_9_ phase could be TiO_2_-excess compared to La_2_Ti_3_O_9_, as the La_2_Ti_2_O_7_ composition is TiO_2_-poor compared to La_2_Ti_3_O_9_, the end member of LLT (*x* = 0.00). First, TSFZ growth was performed using a solvent with 3–6 mol% TiO_2_-excess composition relative to the La_2_Ti_3_O_9_ composition. The La_2_Ti_2_O_7_ phase was detected in the grown crystals when the TiO_2_-excess composition solvent was used, and deposition of the La_2_Ti_2_O_7_ phase was not suppressed. Next, 5 or 10 mol% La_2_Ti_2_O_7_-poor composition solvents relative to the La_2/3-*x*_Li_3*x*_TiO_3_ (*x* = 0.117) composition were used. For those solvents, figure 2 shows photographs of the as-grown crystals and concentration mapping images of La and Ti in the cross-section parallel to the growth direction. Compared with the FZ growth case, the crystal growth stabilized when the solvent was used. Hence, LLT crystals of approximately 5 mm*φ* and 20 mm in length were obtained. The concentration mapping image of the crystal obtained using the 5 mol% La_2_Ti_2_O_7_-poor solvent shown in figure 2*a* indicates that the La_2_Ti_2_O_7_ phase was deposited as an inclusion in the initial growth region. By contrast, the La_2_Ti_2_O_7_ phase was not detected in the crystal grown using the 10 mol% La_2_Ti_2_O_7_-poor solvent, as shown in figure 2*b*. Therefore, inclusion-free LLT crystals were grown using the solvent with 10 mol% La_2_Ti_2_O_7_-poor composition relative to the stoichiometric LLT (*x* = 0.117).

### 3.3. Molten zone stabilization through feeding and growth rate control

Once the habit planes were formed, the melt in the molten zone tended to spill outward, because of the fluctuation of the mass balance between the feed and crystallization volumes. To maintain the molten zone stability for a long period of time, the balance between the feed and growth rates was investigated.

**Figure 3.**
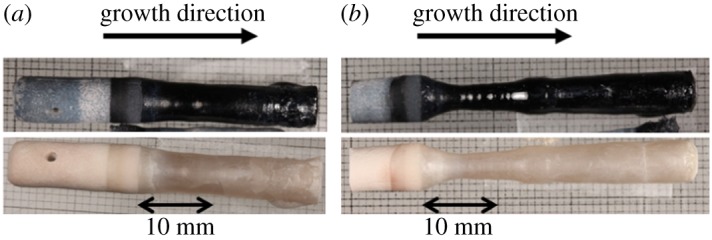
Grown (upper) and annealed (lower) crystals obtained at different feed rates: (*a*) 5 and (*b*) 3 mm h^−1^.

**Figure 4.**
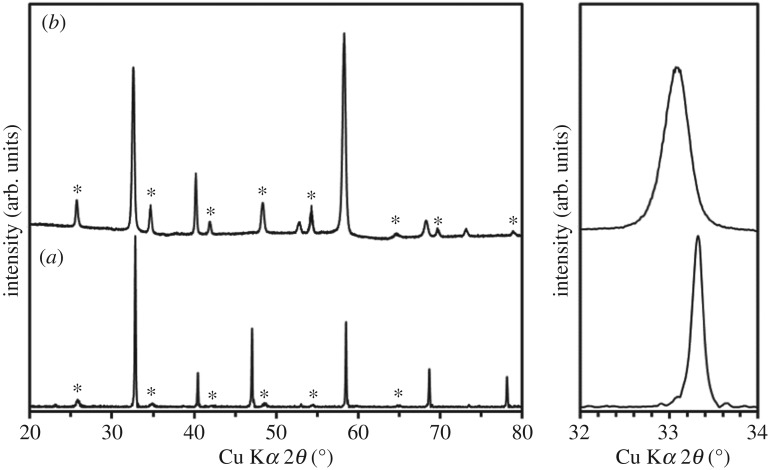
XRD patterns of (*a*) the feed rod sintered at 1300°C for 12 h in air and (*b*) the grown crystals. The asterisks * in the XRD patterns indicate the diffraction peaks corresponding to a superstructure in the double-perovskite structure of LLT.

To control the mass balance between feeding and crystallization, the feed rate was changed with respect to the 5 mm h^−1^ growth rate. When the feed rate was identical to the 5 mm h^−1^ growth rate, the molten zone volume became large, and the melt near the interface between the feed and crystal formed a bulge. It seems that a feed oversupply occurred, which limited the LLT crystal growth to 20 mm in length because of the melt drop. By contrast, a suitably large molten zone was stably maintained for a long period of time by decreasing the feed rate to 3 mm h^−1^. Figure 3 shows the grown crystals and the crystals annealed in oxygen. LLT crystals of approximately 5 mm*φ* and 37 mm in length were obtained by controlling the feed rate relative to the growth rate. Again, note that black crystals were obtained because of oxygen vacancy in the crystals, as the crystals were grown in an Ar gas flow. However, when the grown crystals were annealed in an oxygen gas flow, they became colourless. The chemical composition of the grown crystals was determined to be La_0.61±0.02_Li_0.17±0.006_TiO_3_ (*x* = 0.057 ± 0.002) by quantitative analysis using EPMA. The Li content in the grown crystals was lower than that in the feed. The low Li content is due to the evaporation of Li from the molten zone during the crystal growth and a lower distribution coefficient of Li into LLT. Figure 4 shows the XRD patterns of the LLT feed rod sintered at 1300°C for 12 h in air and the grown crystals. The XRD pattern of the grown crystals is attributed to a tetragonal double-perovskite-type LLT. Thus, we successfully obtained double-perovskite-type LLT crystals by the TSFZ method. The diffraction peaks of the grown crystals shifted to lower angle than those of the feed rod. It had been reported that the LLT lattice expands with the extra Li evaporation [21]. The peak shift in the grown crystals agrees with the results of the quantitative analysis by EPMA. The diffraction intensity due to the superstructure in the grown crystals was high compared with that of the sintered LLT crystals. This indicates that the grown crystals have a high crystallinity. Cracks due to annealing were observed in those crystals. In future work, crack-free LLT crystal growth is expected through optimization of the growth conditions, such as the Li concentration and growth rate.

The crystallographic axes were identified by the back-reflection Laue X-ray diffraction method and X-ray diffraction analysis was performed with a 2D detector. The growth direction and habit planes were [001] and (110), respectively.

### 3.4. Ionic conductivity of LLT crystals

**Figure 5.**
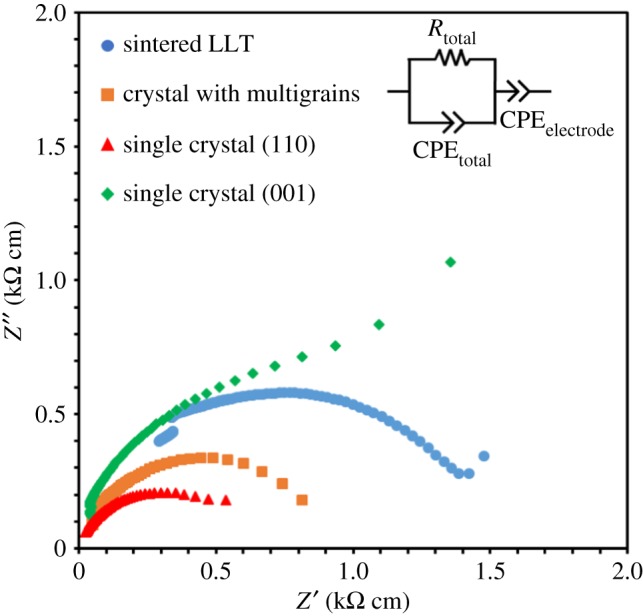
Impedance plots of LLT samples at room temperature. The crystals were annealed in oxygen.

Figure 5 shows the complex AC impedance plots for [110] and [001] in the LLT single crystals, sintered LLT and an LLT crystal with multigrains at room temperature, where the crystal samples annealed in oxygen were used for the measurement. A semicircular trend is observed at a higher frequency range, whereas a straight line is apparent at a lower frequency range. It is difficult to separate the bulk resistance and grain boundary resistance because only one semicircle is observed. In the impedance plots, the semicircle represents the total resistance of the solid electrolyte. The straight line is attributable to the electrode polarization in the blocking electrodes. The total resistance of the sample was estimated from the crossing point of the extrapolated semicircle with the real axis in the lower frequency range.

The *σ* values of the samples are listed in table 1. The ionic conductivities along [110] and [001] in the LLT single crystals were *σ*[110] = 1.64 × 10^−3^ S cm^−1^ and *σ*[001] = 5.26 × 10^−4^ S cm^−1^, respectively. The anisotropic ionic conductivity of the grown crystal was *σ*[110]/*σ*[001] ≈ 3. It has been reported that double-perovskite-type LLT is composed of alternately stacked La-rich and -poor layers [20]. A large amount of Li^+^ occupies the La^3+^ sites in the La-poor layer [20], and the Li^+^ ions can move quickly within the La-poor layer because of the high Li^+^ concentration. However, the Li^+^ migration tends to be blocked by La^3+^ cations in the La-rich layer [20,29], suggesting that Li^+^ ions can more easily migrate along [110] than [001]. The *σ* in the sintered LLT was 6.90 × 10^−4^ S cm^−1^, slightly higher than that along [001] in the LLT single crystals. This finding suggests that the mobility of Li^+^ along [001] in LLT is a determining factor of the *σ* in LLT. The *σ* in the LLT crystal with multigrains was 1.18 × 10^−3^ S cm^−1^, twice that of the sintered LLT. Thus, a high *σ* of 10^−3^ S cm^−1^ was obtained in the crystals grown by the TSFZ method. We are planning to investigate the relationship between the Li content of the grown crystals and their *σ* values in future work, by considering the Li concentration within the LLT crystals.

**Table 1.** Ionic conductivities of LLT samples at room temperature.

sample	ionic conductivity *σ* (S cm^−1^)
single crystal [110]	1.64 × 10^−3^
single crystal [001]	5.26 × 10^−4^
crystal with multigrains	1.18 × 10^−3^
sintered LLT	6.90 × 10^−4^

## 4. Conclusion

In this study, LLT was confirmed to be an incongruent-melting compound and larger LLT single crystals were grown by the TSFZ method. The incongruent melting of LLT was confirmed because the La_2_Ti_2_O_7_ phase was deposited as a primary phase in the initial growth region during FZ growth of LLT crystals. Although LLT crystals of approximately 4 mm*φ* and 11 mm in length were obtained through the FZ method, it was difficult to maintain the stable molten zone for a long period of time because of the inhomogeneous dissolution of the feed rod. For TSFZ growth of LLT, inclusion-free LLT single crystals were obtained when a solvent of 10 mol% La_2_Ti_2_O_7_-poor composition relative to the stoichiometric LLT was used. By contrast, a solvent with 5 mol% La_2_Ti_2_O_7_-poor composition relative to the stoichiometric LLT caused precipitation of the La_2_Ti_2_O_7_ phase as an inclusion in the grown crystals. Moreover, the molten zone was stably maintained for long period of time by decreasing the feed rate from 5 to 3 mm h^−1^. Hence, LLT crystals of approximately 5 mm*φ* and 37 mm in length were obtained by the TSFZ method. Anisotropic ionic conductivity of *σ*[110]/*σ*[001] ≈ 3 at room temperature was observed in the LLT single crystals, with *σ*[110] = 1.64 × 10^−3^ S cm^−1^ and *σ*[001] = 5.26 × 10^−4^ S cm^−1^. LLT single crystals orientated parallel to the *c*-axis are expected to be candidates for high-performance solid-state electrolytes in all-solid-state Li ion batteries.

## Supplementary Material

Reviewer comments

## Data accessibility

We have already included all data within the manuscript.
